# Plasma proteomics profiles predict the risk of future aortic aneurysm and aortic dissection

**DOI:** 10.1097/JS9.0000000000002845

**Published:** 2025-06-27

**Authors:** Maohua Li, Xiao He, Wen Gong, Shasha Xiao, Keyun Fu, Qi Qin, Lunchang Wang, Xin Li, Chang Shu, Jiehua Li, Zhaowei Zhu

**Affiliations:** a Department of Vascular Surgery, The Second Xiangya Hospital of Central South University, Changsha, China; b Molecular Biology Research Center, School of Life Sciences, Hunan Province Key Laboratory of Basic and Applied Hematology, Central South University, Changsha, China; c Department of Cardiology, The Second Xiangya Hospital of Central South University, Changsha, China; d Institute of Vascular Diseases, Central South University, Changsha, China; e Center of Vascular Surgery, Fuwai Hospital, National Center for Cardiovascular Disease, Chinese Academy of Medical Sciences and Peking Union Medical College, Beijing, China

**Keywords:** aortic aneurysm, aortic dissection, plasma proteomics, risk prediction, UK biobank

## Abstract

**Background:**

Aortic aneurysms and aortic dissections (AA/AD) are serious vascular conditions that often progress without symptoms and are associated with high mortality, highlighting the need for improved tools to predict the occurrence. This study aims to identify plasma proteins that can predict the risk of future AA/AD events and to combine these biomarkers with traditional risk factors to construct risk prediction model.

**Materials and methods:**

We analyzed plasma proteomic data from 22 416 participants in the UK Biobank, measuring 2911 proteins using the Olink Explore proximity extension assay. Plasma proteomics data were analyzed using Cox regression and machine learning techniques. Proteins significantly associated with AA/AD risk were identified, and predictive models were constructed by integrating these biomarkers with traditional risk factors such as age, sex, and blood pressure.

**Results:**

The Cox regression models identified 25 proteins significantly associated with AA/AD risk, after adjusting for demographic factors. Furthermore, light gradient-boosting machine was used to rank the importance of these proteins and applied forward stepwise selection to identify four key predictive proteins (cystatin 3 [CST3], matrix metallopeptidase 12 [MMP12], multiple EGF-like domains 9 [MEGF9], and C-X-C motif chemokine ligand 17 [CXCL17]). The protein panel demonstrated an overall predictive AUC of 0.725 for AA/AD. The demographic model achieved an AUC of 0.740. Integration of these biomarkers with demographic factors significantly enhanced predictive accuracy, achieving an AUC of 0.777 (DeLong test *P*<0.001). Temporal trajectory analysis revealed that elevated levels of CST3, MMP12, and CXCL17 were detectable up to 10 years prior to AA/AD diagnosis.

**Conclusion:**

Our study highlights the potential of plasma proteomics, particularly combination of four proteins (CST3, MMP12, MEGF9, and CXCL17), as a valuable strategy for predicting AA/AD risk. The integration of proteomic biomarkers with demographic factors enhances predictive accuracy and offers insights into the underlying molecular mechanisms, which could lead to improved early detection and personalized treatment for AA/AD.

## Introduction

Aortic aneurysms and aortic dissections (AA/AD) are severe vascular conditions characterized by pathological dilation or tearing of the aortic wall, which can lead to catastrophic complications such as rupture, organ ischemia, or death^[1-3]^. Aortic aneurysms are the fifteenth leading cause of death in individuals aged ≥55 years, with an increasing incidence rate as age advances and a higher incidence in men than in women. The incidence of aortic dissection is approximately 3.4 cases per 100 000 person-years^[4,5]^. Despite advances in surgical and endovascular treatment, the asymptomatic nature of these diseases in their early stages often delays diagnosis and intervention, highlighting the need for improved tools to predict their occurrence^[6-10]^.

Risk stratification for AA/AD has traditionally relied on established clinical and demographic risk factors, such as age, male sex, hypertension, smoking, and family history of cardiovascular disease (CVD)^[11–13]^. Traditional risk scores based on traditional risk factors for CVD provide at best a modest degree of differentiation[14]. Proteomics has made significant progress in predicting CVDs and shows great potential^[15,16]^. Currently, widely used plasma biomarkers, such as troponins and natriuretic peptides, only cover a limited range of CVD pathways and fail to comprehensively reflect the complexity of these conditions^[17,18]^. By systematically analyzing the plasma proteome, proteomics offers opportunities for unbiased discovery of novel biomarkers, which not only enhance diagnostic and predictive accuracy but also provide insights into pathophysiological mechanisms and potential therapeutic targets[17]. Recent technological advancements have overcome the challenges posed by the complexity of the human plasma proteome, such as the use of nucleotide-labeled immunoassays, aptamer reagents, and machine learning techniques. These innovations have greatly improved sensitivity, specificity, and throughput in protein detection^[17,18]^. Furthermore, high-throughput proteomics combined with machine learning has shown superior performance in risk stratification compared to traditional clinical models. For example, a proteome-based risk model for recurrent atherosclerotic CVD outperformed clinical models (AUC: 0.810 vs. 0.750) and identified neutrophil-related pathways, suggesting the presence of residual inflammatory risk beyond traditional pathways[19]. Overall, proteomics not only facilitates the discovery of novel cardiovascular biomarkers but also provides a scientific foundation for personalized therapeutic and preventive strategies.

The UK Biobank, a large-scale prospective cohort study with over 500 000 participants, is an invaluable resource for exploring the relationship between plasma proteomics and aortic disease risk[20]. By integrating proteomic profiles with longitudinal clinical outcomes, the UK Biobank offers the opportunity to identify novel biomarkers and pathways associated with the future development of AA/AD. Previous studies using the UK Biobank have demonstrated the value of proteomic data in predicting various CVDs, highlighting its potential for this application[21].

This study aims to use the extensive proteomic dataset from the UK Biobank to identify plasma proteins associated with the future risk of AA/AD. By combining proteomic data with demographic factors, we seek to construct predictive models that can stratify individuals by their likelihood of developing these conditions. Ultimately, this research could enhance our understanding of the molecular mechanisms underlying aortic disease, facilitate earlier diagnosis, and guide targeted prevention and management strategies.

## Materials and methods

### Study population/study design

**Figure 1.**
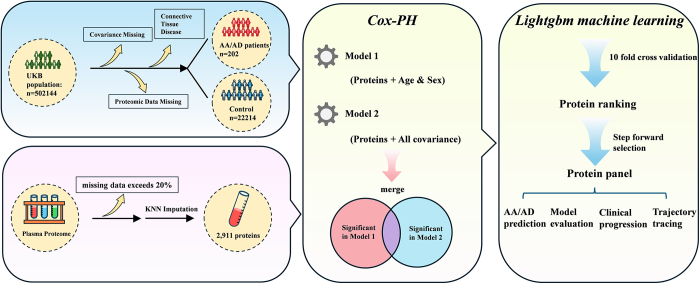
Flowchart of the study. Plasma proteomic data from 22 416 participants free of AA/AD at baseline were analyzed. Cox proportional hazards regression models were employed to identify proteins associated with AA/AD risk. Proteins exhibiting significant associations in both Model 1 (adjusted for age and sex) and Model 2 (adjusted for multiple factors) were selected. The LightGBM machine learning algorithm was utilized to rank the importance of these proteins, and forward stepwise selection was applied to identify key predictive proteins. The temporal trajectories of these proteins were examined to assess their changes prior to AA/AD diagnosis. And proteomic biomarkers were integrated with demographic factors to construct a risk prediction model.

The study population included participants from the UK Biobank who were randomly selected for plasma proteomics analysis during the baseline visit. Baseline blood samples were collected during the period from 2007 to 2010. Subsequently, standardized quantitative processing was carried out using the antibody-based Olink Explore proximity extension assay. The detailed protocols for sample processing, plasma analysis using the Olink proteomics detection method, data processing, and quality control procedures have been described in previous publications[22]. For the proteomics data, proteins with more than 20% missing values across the subjects were excluded, leaving 2911 proteins for further analysis. Missing values were imputed using the K-nearest neighbors method[23]. For analytical purposes, participants with baseline AA/AD, connective tissue diseases, or missing data on relevant covariates were excluded. This resulted in an analytical cohort of 22416 participants (median age 57 years, 49.55% female) (Fig. 1). The work was reported in line with the STROCSS criteria^[24,25]^. And it was registered in www.researchregistry.com with a registration unique identifying number (UIN) as researchregistry11273 (https://www.researchregistry.com/browse-the-registry#home/).

**HIGHLIGHTS**

- A series of 25 proteins were identified to be significantly associated with future risk of aortic aneurysm and aortic dissection (AA/AD).
- A panel of four proteins (CST3, MMP12, MEGF9, and CXCL17) was demonstrated to be a valuable strategy for predicting AA/AD risk.
- The integration of proteomic biomarkers with demographic factors further enhances predictive accuracy for future AA/AD.

### AA/AD outcomes

The primary endpoint was the incidence of AA/AD (International Classification of Diseases, 10th Revision [ICD-10] I71), defined as the combined outcome of AA (ICD-10 I71.1–I71.9) and AD (ICD-10 I71.0). Reported cases of AA/AD were identified through sources such as death registries, primary care records, hospital data, and self-reported diagnoses. The onset date for both conditions was defined as the date of the first AA/AD report. Participants were followed from baseline until the date of AA/AD diagnosis, date of death, or the end of the follow-up period (6 July 2024), whichever occurred first[26].

### Demographic factors

Baseline characteristics were collected at the baseline examination. The covariances used in the models including age, sex, family income, Townsend deprivation index, education, employment, drinking status, health status, healthy diet score, physical activity, body mass index, estimated glomerular filtration rate (GFR), systolic blood pressure, glycated hemoglobin, lipid profile, antihypertensive medication, anti-hyperglycemic medication, and lipid-lowering medication[27]. The specific definitions of the relevant covariates and the corresponding UKB field IDs can be found in Supplementary Table 1, http://links.lww.com/JS9/E501.

**Table 1:** Population characteristics at baseline and incident endpoints

Characteristics	Incident AA/ADs		Whole cohort (n = 22 416)	
	Yes (n = 202)	No (n = 22 214)		*P* value
Age*	62(58-67)	57(49-63)	57(49-63)	2.35^a^10^−18^
Female sex*	29(14.4%)	11 079(49.9%)	11 108(49.6 %)	1.02^a^10^−22^
BMI*	27.8(25.2-31.0)	26.6(24.1-29.5)	26.6(24.1-29.6)	1.21^a^10^−4^
Diet score	5(5-6)	5(5-6)	5(5-6)	0.91
Glycated hemoglobin*	35.4(33.2-38.4)	35(32.5-37.4)	35(32.5-37.4)	0.04
Total cholesterol*	5.3(4.4-6.2)	5.6(4.9-6.4)	5.6(4.9-6.4)	3.96^a^10^−5^
HDL*	1.2(1.1-1.5)	1.4(1.2-1.7)	1.4(1.2-1.7)	7.39^a^10^−13^
LDL*	3.2(2.7-4.0)	3.5(2.9-4.1)	3.5(2.9-4.1)	3.02^a^10^−3^
Triglycerides*	1.8(1.2-2.4)	1.5(1.0-2.1)	1.5(1.0-2.1)	4.07^a^10^−5^
eGFR*	90.7(86.0-96.4)	95.8(90.3-101.4)	95.7(90.3-101.4)	2.52^a^10^−14^
Systolic blood pressure*	136(125-146)	132(121-145)	132(121-145)	8.78^a^10^−3^
TDI	−2.165(−3.905-0.185)	−2.42(−3.78–0.04)	−2.42(−3.78–0.03)	0.53
Antihypertensive medication*	76(37.6%)	2551(11.5%)	2627(11.7%)	5.26^a^10^−29^
Antihyperglycemic medication	1(0.5%)	129(0.6%)	130(0.6%)	1.00
Lipid lowering medication*	72(35.6%)	2440(11.0%)	2512(11.2%)	4.96^a^10^−27^
Physical activity	120(60-198.8)	100(60-175)	100(60-175)	0.05
Current smoking*	33(16.3%)	1894(8.5%)	1927(8.6%)	6.80^a^10^−6^
Current alcohol	195(96.5%)	20 972(94.4%)	21 167(94.4%)	0.25
In paid employment*	90(44.6%)	13 719(61.8%)	13 809(61.6%)	8.57^a^10^−9^
Higher income	61(30.2%)	7423(33.4%)	7484(33.4%)	0.43
Higher education*	57(28.2%)	8253(37.2%)	8310(37.1%)	0.02

Values are given as median (interquartile range), or n (%). BMI, body mass index; HDL, high-density lipoprotein; LDL, low-density lipoprotein; eGFR, estimated glomerular filtration rate; TDI, Townsend deprivation index.

Mann–Whitney U for continuous variables, X2 for categorical variables; Benjamini–Hochberg correction for multiple testing.

* *P*<0.05.

### Cox regression analysis

Cox proportional hazard regression models based on follow-up time scales were used to estimate the hazard ratio (HR) and the corresponding 95% confidence interval for the association between circulating protein and the risk of AA/AD development. Two models were used: (a) Model 1 was adjusted for sex and age; (b) Model 2 was adjusted for age, sex, family income, Townsend deprivation index, education, employment, drinking status, health status, healthy diet score, physical activity, body mass index, estimated GFR, systolic blood pressure, glycated hemoglobin, lipid profile, antihypertensive medication, anti-hyperglycemic medication, and lipid-lowering medication. The BH correction was applied to account for multiple tests. A *P*-value <0.05 was considered statistically significant.

### Machine learning

The purpose of machine learning is to compare the relative importance of identified protein biomarkers using a model with minimal assumptions. The relative importance of the identified proteins was examined using the light gradient-boosting machine (LightGBM).The protein’s predictive performance was evaluated by using tenfold cross validation[28]. A sequential forward selection method was applied, in which proteins were added to the newly developed LGBM one at a time, in order of their importance. Once the optimal performance of AUC is achieved, the selection process terminates, and the optimal performance of AUC is defined as incremental performance not detected in two consecutive DeLong tests. Shapley additive explanations (SHAP) were then used to estimate the average contribution of each predictor to the overall model prediction[29].

### Ethics statement

This study adheres to the Helsinki Declaration. Before participation, all subjects provided written consent and received approval from the Northwest Multi-center Research Ethics Committee (11/NW/0382)^[22,30]^. We extend our heartfelt gratitude to all the participants of the UK Biobank and everyone who contributed to the development of the UK Biobank study. This research was conducted using the UK Biobank Resource under Application Number 84709.

## Results

### Identifying proteins associated with AA/AD

**Figure 2.**
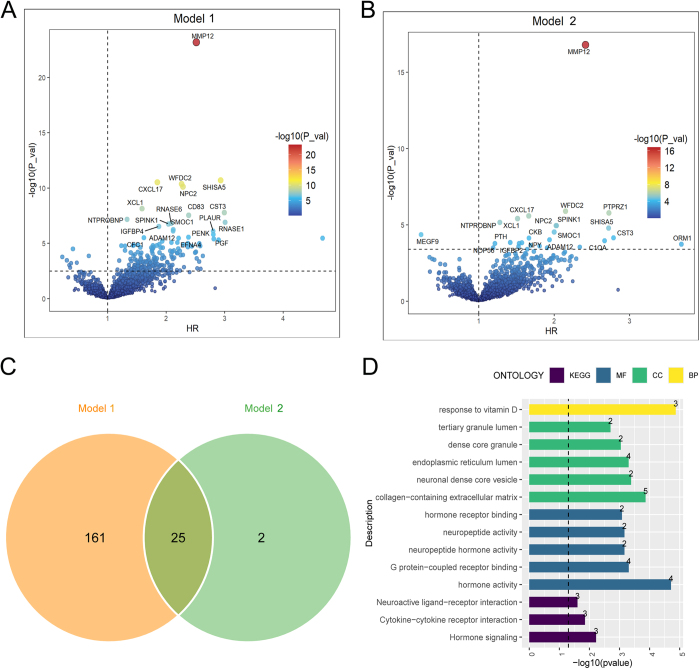
Associations of plasma proteins with AA/AD. The volcano plot shows the associations of 2911 proteins with AA/AD in Model 1. Hazard ratios (HR) are shown on the x-axis and -log10(*P*-values) on the y-axis. Proteins above the horizontal dashed line (BH-corrected *P*<0.05) represent significant associations. (B) Corresponding volcano plot for Model 2, using identical axes and significance thresholds as Panel A. (C) The Venn diagram shows proteins significantly associated with AA/AD in both models. (D) Enrichment analysis for gene ontology (GO) and the Kyoto Encyclopedia of Genes and Genomes (KEGG) was performed using the R package cluster Profiler for the significant proteins in in both models. Statistical significance was defined as *P*-value <0.05 (vertical dashed line). Bar labels show protein counts per pathway. BP, biological process; CC, cellular component; MF, molecular function.

This study included 22 416 adults without AA/AD at baseline, with a median age of 57 years, of whom 11 108 (49.55%) were female. During a median follow-up period of 15.3 years, 202 AA/AD events were identified. Table 1 provides detailed information about the participants in this study. Among the 2911 plasma proteins included in the analysis, 186 proteins were found to be significantly associated with AA/AD in Model 1, which was adjusted for age and sex, after BH correction (Fig. 2A, Supplementary Table 2: http://links.lww.com/JS9/E502). As a sensitivity analysis, Model 2 was further adjusted for age, sex, family income, Townsend deprivation index, education, employment, drinking status, health status, healthy diet score, physical activity, body mass index, estimated GFR, systolic blood pressure, glycated hemoglobin, lipid profile, antihypertensive medication, anti-hyperglycemic medication, and lipid-lowering medication. After BH correction, 27 proteins were found to be significantly associated with AA/AD in Model 2 (Fig. 2B, Supplementary Table 3: http://links.lww.com/JS9/E503). Furthermore, a Venn diagram demonstrated that 25 proteins were significantly associated with AA/AD in both Model 1 and Model 2 (Fig. 2C). Among these, matrix metallopeptidase 12 (MMP12) exhibited the strongest association in both models (Model 1: HR = 2.516, *P* = 1.95 × 10⁻^20^; Model 2: HR = 2.416, *P* = 4.77 × 10⁻^14^). Other proteins significantly associated with AA/AD including C-X-C motif chemokine ligand 17 (CXCL17) (Model 1: HR = 1.852, *P* = 3.02 × 10⁻^8^; Model 2: HR = 1.664, *P* = 1.91 × 10⁻^3^), WAP four-disulfide core domain protein (WFDC2) (Model 1: HR = 2.265, *P* = 3.35 × 10⁻^8^; Model 2: HR = 2.152, *P* = 1.63 × 10⁻^3^), and cystatin 3 (CST3) (Model 1: HR = 2.996, *P* = 7.13 × 10⁻^6^; Model 2: HR = 2.789, *P* = 1.69 × 10⁻^3^). These findings suggest that higher levels of MMP12, CXCL17, WFDC2, and CST3 are associated with an increased risk of AA/AD. Additionally, multiple EGF-like domains 9 (MEGF9) were identified to be consistently associated with a decreased risk of AA/AD in both Model 1 (HR = 0.303, *P* = 1.15 × 10⁻^2^) and Model 2 (HR = 0.237, *P* = 1.17 × 10⁻^2^). Finally, enrichment analysis was performed on the 25 proteins that were significantly associated with AA/AD in both models. The results showed that these proteins were enriched in pathways such as response to vitamin D, collagen-containing extracellular matrix, hormone activity, cytokine–cytokine receptor interaction (Fig. 2D, Supplementary Table 4: http://links.lww.com/JS9/E504).

### Protein importance ranking

**Figure 3.**
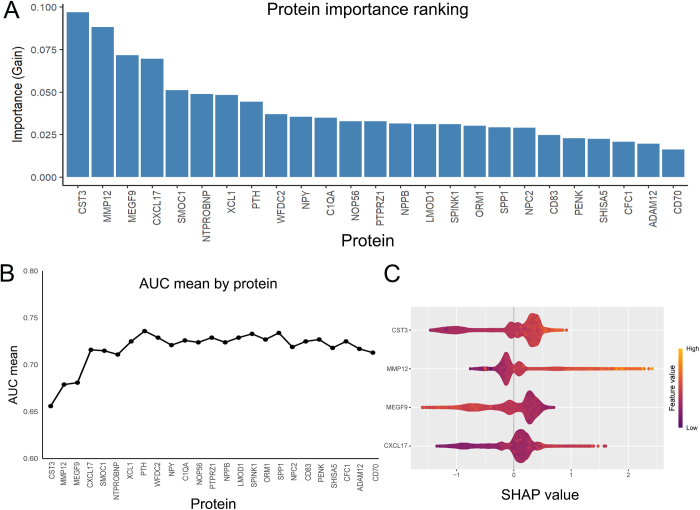
Protein importance ranking with light gradient-boosting machine (LightGBM) and visualization of selected proteins with Shapley additive explanations (SHAP). Protein importance ranking using LightGBM model. The bar chart illustrates the importance of proteins categorized based on their contribution to predicting future AA/AD (determined by information gain). (B) A line plot showing the cumulative AUC value changes calculated using the stepwise forward selection method. The x-axis represents the number of proteins included, and the y-axis represents the specific cumulative AUC value. (C) SHAP visualization of selected proteins. The width of the horizontal bars represents the degree of contribution to AA/AD prediction: the wider the range, the greater the contribution. The direction on the x-axis indicates the likelihood of developing AA/AD (right) or not (left).

Proteins significantly associated with AA/AD in both Model 1 and Model 2 were analyzed using a LightGBM model to determine their relative importance (Fig. 3A). Detailed results are provided in Supplementary Table 5: http://links.lww.com/JS9/E505. Based on gain ranking, CST3 was identified as the top-ranking protein in importance, demonstrating the strongest association with AA/AD. MMP 12 and CXCL17 also ranked highly, consistent with their significant associations in both Model 1 and Model 2. We then applied a stepwise forward selection method, progressively adding proteins to the model in decreasing order of importance and calculating the AUC at each step. As the number of proteins increased, the AUC value sharply rose initially and then gradually stabilized. The top four proteins (CST3, MMP12, MEGF9, and CXCL17) were selected for subsequent analysis (Fig. 3B). And we plotted SHAP values to visualize how the selected proteins contributed to AA/AD prediction. The SHAP plot illustrated the influence of each protein based on its numerical value (encoded with a color gradient) and its effect along the horizontal axis (representing the likelihood of developing AA/AD) (Fig. 3C). Among the four proteins, MMP12 showed the widest SHAP value distribution among all AA/AD cases, indicating its robust predictive performance.

### Predictive accuracy of plasma proteins

**Figure 4.**
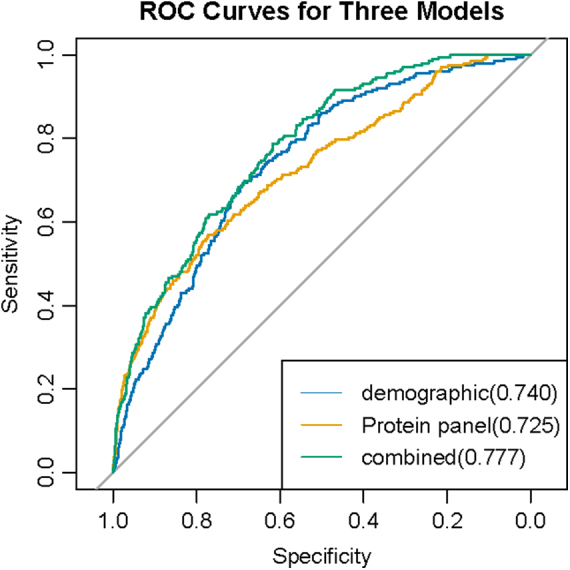
Predictive performance of three models. Predictive performance assessment of three models: (i) model with demographics only, (ii) model with protein panel only, and (iii) model with protein panel and demographics combined.

To evaluate the predictive accuracy of the identified proteins, we employed 10-fold cross-validation to calculate their AUC values. For AA/AD events, the individual plasma proteins exhibited the following mean AUC values: CST3 (0.667), MMP12 (0. 668), MEGF9 (0.543), CXCL17 (0.652) (Supplementary Table 6: http://links.lww.com/JS9/E506). The AUC of the protein panel composed of four proteins reached 0.725, highlighting their potential for predicting AA/AD. The model with only the demographic factor has an AUC value of 0.740 (Fig. 4). To further improve the model’s performance, we added a protein panel consisting of four proteins. When combined with protein panel, the AUC values of individual proteins were significantly improved. Notably, the combination of the four proteins with clinical covariates achieved an AUC as high as 0.777 (DeLong test *P* = 7.76 × 10^⁻5^) (Fig. 4)). These findings suggest that our composite model, which incorporates both protein biomarkers and clinical information, could predict the future occurrence of AA/AD events more accurately.

### Plasma proteins and the risk of clinical progression

**Figure 5.**
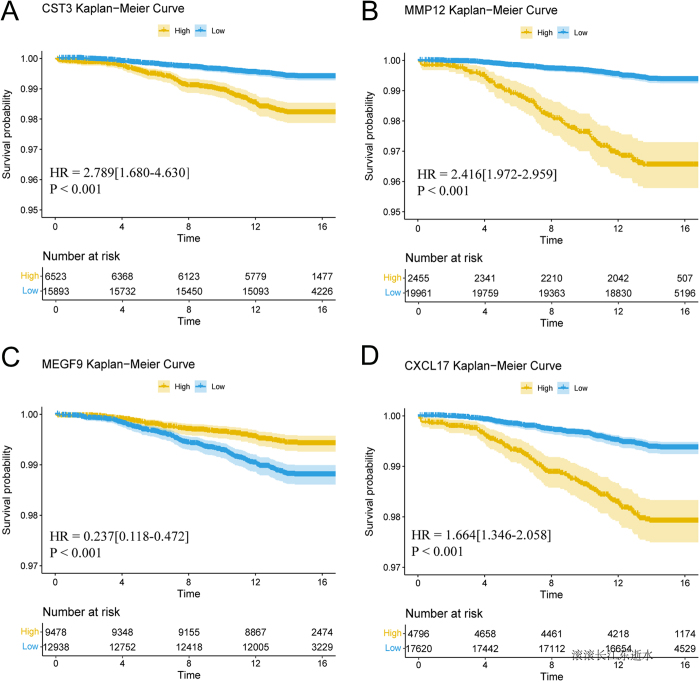
Predictive performance of baseline protein levels for clinical progression risk. Kaplan–Meier curves present the clinical progression to AA/AD over time, visualized for individuals with low (blue line) and high (red line) baseline plasma CST3 (A), MMP12 (B), MEGF9 (C), CXCL17 (D) levels.

We then evaluated the prognostic value of baseline plasma proteins in the progression to AA/AD. Baseline protein levels were divided into high and low groups, with the cutoff point determined using the surv cutpoint function in the R package survminer, which differentiated those who experienced clinical progression throughout the follow-up period from those who did not. Elevated baseline levels of CST3, MMP12, and CXCL17 were associated with significantly increased AA/AD risk (Fig. 5A, B, D). Conversely, elevated MEGF9 levels showed a protective association with reduced AA/AD risk (Fig. 5C). Notably, individuals with higher CST3 levels had a 3.0-fold higher likelihood of developing AA/AD compared to those with lower baseline CST3 levels. Similarly, individuals with higher MMP12 levels had a 2.4-fold higher likelihood of developing AA/AD in the future compared to those with lower levels.

### Trajectories of plasma proteins before diagnosis

**Figure 6.**
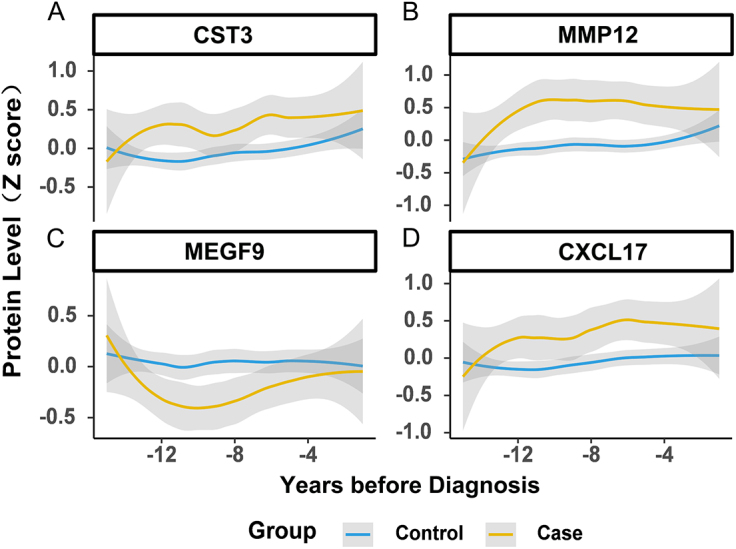
Longitudinal protein profile changes preceding AA/AD diagnosis. The dynamic changes in plasma levels of CST3, MMP12, MEGF9, and CXCL17 before the diagnosis of AA/AD were plotted. A nested case–control study was conducted, where an individual with an incident event (within a 15-year observation period) was matched (with five healthy controls based on matching criteria of age and sex).

Finally, we analyzed the longitudinal trajectories of selected plasma proteins over a 15-year pre-diagnosis period, comparing AA/AD cases with matched controls. Figure 6 displays protein level dynamics during the 15-year pre-diagnosis window. CST3 levels were significantly elevated in eventual AA/AD cases versus controls up to 10 years before diagnosis and remained elevated thereafter. Similar trends were observed for MMP12 and CXCL17 (Fig. 6A, B, D). While MEGF9 exhibited an inverse temporal pattern (Fig. 6C), MEGF9 levels were consistently lower in AA/AD cases, displaying a characteristic inverted U-shaped trajectory distinct from controls.

## Discussion

Aortic aneurysms and aortic dissections (AA/AD) pose significant clinical challenges due to their high mortality and morbidity, often presenting at advanced stages when interventions are less effective^[31,32]^. The asymptomatic progression of these diseases necessitates the identification of reliable biomarkers for early detection and risk prediction^[33–35]^. In this study, we used the extensive plasma proteomic data from the UK Biobank to identify proteins associated with the future rigs and suggest that these proteins may play critical roles in the pathophysiology of aortic diseases, particularly in immune-related pathways. Integrating these biomarkers with demographic factors enhanced predictive accuracy, achieving an AUC of 0.777, which is a substantial improvement compared to models using proteins alone. Temporal trajectory analysis also revealed that elevations in CST3, MMP12, and CXCL17 occurred up to 10 years prior to the diagnosis of AA/AD, underscoring their potential for early detection. These findings align with previous research implicating similar biomarkers in vascular remodeling and inflammation that contribute to the development of AA and AD.

Cystatin C (CST3) is a protease inhibitor secreted by all nucleated cells and is widely used for estimating GFR. It is more sensitive than creatinine in detecting early renal dysfunction as it is not influenced by muscle mass or gender[36]. In recent years, its predictive value in CVD has gained significant attention. Observational studies have shown that elevated CST3 levels are strongly associated with coronary artery disease, ischemic stroke, heart failure, and all-cause mortality. For instance, in elderly men, CST3 was independently associated with diabetes, obesity, inflammatory markers (such as hsCRP), and cardiovascular events[37]. Long-term follow-up studies have further confirmed its ability to predict cardiovascular mortality in patients with acute coronary syndrome over a 16-year period[38]. Notably, CST3 plays a prominent role in cardiovascular risk stratification in patients with chronic kidney disease, with superior predictive ability compared to creatinine, especially in those with mildly reduced GFR[39]. However, a Mendelian randomization study[40] found that although CST3 gene variants (such as rs911119) reduced serum levels by 6.13%, it did not alter the risk of CVD (HR = 1.00), suggesting that the observed associations may be due to residual confounding (such as inflammation or vascular injury) rather than causal relationships. Furthermore, although the CST3 gene promoter haplotype (−82 G/-5 G/ + 4A) affects plasma levels, it was not directly associated with cardiovascular events[41]. Meta-analyses further indicated that each standard deviation increase in CST3 was associated with a 57% increased risk of cardiovascular mortality^[42,43]^. Our study further suggests that CST3 holds significant value as a risk marker for AA/AD, although its potential as a therapeutic target remains to be validated. Matrix metalloproteinase 12, also known as human macrophage elastase, plays a key role in the pathogenesis of AAA. Studies have shown that MMP12 is highly expressed in AAA tissues, particularly in infiltrating macrophages, and is localized to areas of elastin degradation in the aortic wall[44]. Circulating biomarkers related to extracellular matrix degeneration, such as MMP12, have been correlated with AA/AD progression, supporting their biological relevance in disease monitoring^[45,46]^. Studies have shown that circulating levels of CXCL17 increase with age and are associated with cardiac dysfunction. C-X-C motif chemokine ligand 17 deletion or neutralizing antibody therapy can significantly inhibit cardiac hypertrophy and fibrosis induced by aging and angiotensin II and improve cardiac function[47]. The research on MEGF9 in the cardiovascular system primarily focuses on its role in sepsis-related cardiac injury, cardiac remodeling, and potential signaling regulation. Studies suggest that MEGF9 protects cardiac function by activating the AMPK pathway, reducing LPS-induced inflammation and oxidative damage[48]. Moreover, MEGF9 may interact with the PI3K/AKT signaling pathway, influencing cardiovascular cell function and playing a potential role in cardiac hypertrophy and vascular remodeling[49]. Its interactions with miR-125b, estimated glomerular filtration rate, and other signaling molecules further indicate its regulatory potential in CVDs^[49,50]^. Additionally, MFGE8, a member of the same family as MEGF9, has been shown to exert a negative regulatory effect in cardiac hypertrophy[51], suggesting that MEGF9 may similarly play a protective role in the cardiovascular system. Although current research is still in the preliminary stages, this study highlights the potential of MEGF9 as a cardiovascular protective factor, offering new strategies for the diagnosis and treatment of CVDs.

Research on predicting AA and AD is currently advancing toward a multidimensional and integrative approach. By combining deep learning with imaging techniques, researchers are now able to quantitatively analyze changes in aortic diameters. Additionally, genome-wide analyses have been employed to identify genetic loci associated with aortic degeneration, providing new biomarkers for disease prediction[52]. And fluid dynamics-based analyses have shown that abnormal aortic dilation is associated with the transition of blood flow from stable laminar flow to unstable aortic fluttering. Predicting AA based on this unstable “fluttering” has shown an accuracy of 98%[53]. In the study of AAA, biomechanical biomarkers, such as wall shear stress and intra-luminal thrombus thickness, have been utilized to predict local growth[54]. Moreover, low shear stress has been found to be associated with the expansion rate and rupture risk of AAA, further highlighting the critical role of hemodynamics in disease progression[55]. Hemodynamic simulations have also indicated that the risk of aortic dissection can be accurately predicted using indicators such as vortex intensity[56].

In recent years, with advancements in technology, there has been a significant improvement in the sensitivity and resolution of plasma proteomics methods. Proteomics has become as a powerful tool for identifying novel biomarkers and exploring disease mechanisms in cardiovascular research^[57–59]^. Traditional risk factors do not fully capture the complex biology underlying AA/AD. For example, hypertension, smoking, and family history of CVD are established risk factors, yet they fail to explain the substantial variability in disease outcomes observed in clinical practice[60]. Proteomics offers an unbiased approach for biomarker discovery, enabling the identification of previously unknown factors that may contribute to disease development and progression^[61,62]^. In our study, the use of LightGBM, allowed for the ranking of proteins by their predictive importance. Our study extends these findings by demonstrating that a proteomic-based model can not only identify biomarkers of AA/AD risk but also improve predictive accuracy when combined with demographic factors, offering a more robust tool for clinical risk stratification. The predictive accuracy of the model was further validated by trajectory analysis, which revealed that changes in plasma protein levels precede the clinical onset of AA/AD. Detecting these preclinical changes could pave the way for earlier interventions and ultimately lead to better patient outcomes.

Integrating proteomic biomarkers with demographic factors holds significant clinical promise for AA/AD. This combined approach offers a more precise tool for identifying individuals at high risk of developing these conditions, particularly those who may be overlooked by traditional risk factor assessments alone. Given the asymptomatic nature of AA/AD in their early stages, this approach could lead to earlier diagnosis and improved patient outcomes by initiating timely interventions. Predicting the risk of AA/AD opens possibilities for targeted surveillance and early intervention strategies. For example, individuals at high risk could be monitored more closely with imaging techniques to assess the condition of the aortic wall, and pharmacological treatments aimed at stabilizing the aorta could be introduced earlier to prevent dissection or rupture. This proactive approach could significantly reduce the morbidity and mortality associated with these conditions. Furthermore, the identification of specific biomarkers, such as CST3, MMP12, and CXCL17, could guide the development of novel therapeutic strategies. The use of biomarkers for monitoring disease progression could also lead to more personalized treatment strategies. Tailoring treatments to the molecular profile of each patient could help clinicians optimize therapeutic outcomes and improve the overall management of AA/AD. Personalized treatment approaches are becoming increasingly important in cardiovascular medicine, as they allow for the consideration of individual patient characteristics, such as genetic predispositions and biomarker levels.

Although our study provides compelling evidence for the role of plasma proteomics in AA/AD risk prediction, there are several limitations that should be addressed. First, Our study was based on UK Biobank data, which on the one hand may limit the generality of our findings to other populations, and on the other hand, participants in UKB were predominantly composed of white patients who were more socioeconomically affluent than the general UK population[63]. Replication of these results in other populations would help to establish the robustness of the biomarkers identified and their applicability to broader clinical settings. Second, although UKB provides a comprehensive assessment of cyclic proteins, not all human proteomes are captured by this platform, and there may be biases in the prioritization of secreted proteins. It is possible to overlook some proteins that are highly associated with CVDs, which may also contribute to AA and AD risk^[29,64]^. Finally, clinical trials are needed to assess whether incorporating of proteomic biomarkers into clinical practice leads to improved patient outcomes. While our study demonstrates the potential of these biomarkers for risk prediction, their practical application in clinical settings requires further validation. Prospective trials that evaluate the impact of proteomic biomarkers on patient outcomes, such as the incidence of aortic rupture or dissection, will be crucial in determining the clinical utility of these markers.

## Conclusion

In this study, we demonstrate that plasma proteomics, when combined with demographic factors, effectively predict the risk of AA/AD. The identification of novel biomarkers, particularly CST3, MMP12, MEGF9, and CXCL17, provides valuable insights into the molecular mechanisms underlying these conditions and opens the door to new therapeutic and risk-stratification strategies. Further validation of these findings in diverse populations and exploration of their clinical utility in longitudinal studies will be crucial in realizing the full potential of proteomics for the early detection and prevention of AA/AD.

## Data Availability

The data in this article will be shared on reasonable request to the corresponding author.
